# Editorial: Mitochondrial therapy in neurodegeneration

**DOI:** 10.3389/fphar.2023.1144093

**Published:** 2023-02-01

**Authors:** Anuradha Kalani, Pankaj Chaturvedi, Dario Brunetti, Komal Kalani

**Affiliations:** ^1^ Chhatrapati Shahu Ji Maharaj University, Kanpur, Uttar Pradesh, India; ^2^ University of Louisville, Louisville, KY, United States; ^3^ University of Milan, Milan, Lombardy, Italy; ^4^ Unit of Medical Genetics and Neurogenetics Fondazione IRCCS Instituto Neurologico Carlo Besta, Milan, Italy; ^5^ University of Texas at San Antonio, San Antonio, TX, United States

**Keywords:** mitochondria, neurodegeneration, Alzheimer’s, Parkinson’s, therapy

For the past decade, mitochondria have been regarded as the target for many therapeutic approaches, against various diseases, such as metabolic, genetic, viral, neurological, or cancer. These therapies, even after going through some clinical trials, have achieved a little success. The mitochondria, being the power house of the cell, *via* generating ATP through the transfer of electrons from complex I to IV, become dysfunctional in various diseases which makes them the target to revive the cells affected by pathological conditions. Neurons, being the master cells and controlling a plethora of metabolic processes, have high energy demand and therefore warrant healthy mitochondria. Owing to the little regenerative capacity of neurons, the diseases that are caused by neuronal dysfunction are often difficult to treat, and scientists along with clinicians are finding every possible way to combat these neurological problems. Mitochondrial therapies represent one of the approaches to revive dysfunctional neurons, thinking that corrected energy metabolism in neurons will render them function properly and promote their regenerative capacity.

Mitochondria become dysfunctional due to a variety of reasons, one of the most common being the reactive oxygen species or ROS. ROS can interfere with the membrane potential of the mitochondria, generated due to the movement of H^+^ ions in return of the ATP molecule generated, and hinder mitochondrial efficiency. Additionally, ROS can independently trigger inflammatory pathways, leading to malfunctioning of the neurons. Most of the therapies have tried antioxidant treatment to resolve ROS which include the use of vitamins C & E, coenzyme Q, N-acetyl-cysteine, catalase derived compounds. The antioxidant treatment also extends toward the use of polypropanoids like quercetin, curcumin, and resveratrol besides the adoption of healthy life styles with diet and exercise to improve neurological function. High-methionine and low-folate diet has been shown to improve cognitive function through mitochondrial function mitigation.

Other reasons for mitochondrial dysfunction include the disruption of electron transport chain (ETC) and ATP uncoupling, opening of the membrane permeability transition pore (MPTP), disruption of the mitochondrial membrane potential, faulty ATP synthases or members of ETC, mutations in the genetic material of mitochondria since mitochondria have circular DNA, and altered ion channels (Ca^+^, Na^+^, and K^+^). The mitochondria that become dysfunctional need to be removed to maintain the mitochondrial dynamics, and this is fulfilled by autophagy, a complex process to engulf the faulty mitochondrial which leads to its degradation and clearance. Since mitophagy being beneficial, it is mediated by a number of pathways that include lipid-mediated, Parkin-mediated, ubiquitin-mediated, and receptor-mediated mitophagy. That is the reason why some of the mitochondrial therapies have targeted mitophagy stimulation by the use of NAD^+^, rapamycin, and iron regulators. The mitochondrial dynamics is also maintained by regulating the mitochondrial fusion by Opa1, Mfn1/2 and fission by Drp1 and Fis1. Drp1 and Mfn2 have also been regarded as the target of mitochondrial therapies, for example, in Alzheimer’s disease (AD). Neurological diseases such as Parkinson’s disease (PD) and AD are underlined by malfunctional mitophagial pathways, thereby altering mitochondrial dynamics.

Other important molecules for mitochondrial therapy include exosomes, which are an emerging branch of drug delivery and when loaded with medicinal molecules like curcumin have been shown to improve neuroregeneration in stroke and traumatic brain injury, by improving mitochondrial function. Some of the genetic therapies for improving mitochondrial function include clustered regularly interspaced short palindromic repeat (CRISPR) approach with mitochondria-specific mitoCas9, mitochondria-based transcription activator-like effector nucleases (TALENs), mtTALENs, and gene therapy by targeting CYP46A1 in case of AD. Drugs such as peroxisome proliferator-activated receptor gamma (PPARγ) agonists or thiazolidinedione drugs (TZDs) and hydralazine have been used in AD pathology that facilitate amyloid *β* plaque clearance and parallelly improve mitochondrial function. Apart from laboratory studies, there have been a significant number of clinical trials that include diet (ketogenic diet NCT04701957, resveratrol with glucose, and malate NCT00678431) and drugs (hydralazine hydrochloride NCT04842552).

The Research Topic of this special issue is committed to the publication of high-quality research and review articles, which may shed light on novel and important mitochondria-based mechanisms and therapeutic interventions in neurodegenerative diseases. In this Research Topic, 31 authors from seven countries have contributed four original research articles and a review, providing important insights into the field of mitochondrial therapy in neurodegeneration. The original research articles submitted include the contributions of Toledo et al., Hu et al., Folbergrova et al., and Hines et al., while the review article includes contribution of Yadav et al.. Toledo et al. have proposed an emerging compound boldine, which is an alkaloid to be effective in AD by clearing the Aβ plaques, thereby interacting with AβO and restoring mitochondrial dysfunction. Hu et al. have investigated the effects of diabetes on mitochondrial dynamic changes in hippocampal neurons and how metformin can mitigate these effects. Folbergrova et al. have demonstrated how resveratrol significantly reduces mitochondrial dysfunction associated with the acute phase of status epilepticus, and the effects of resveratrol are reviewed by Yadav et al. in the review article. Hines et al. have identified that the translocator protein (TSPO) of the outer mitochondrial membrane can serve as a target for therapeutic intervention in psychiatric disorders, by using the TSPO ligands, PK11195, Ro5-4864, and XBD-173 that can alter locomotor behavior and modulate band-specific changes in cortical EEG.

